# Microfluidic Capillary Arrays for Pixelated Control of Multimaterial Flows

**DOI:** 10.1002/smll.202508537

**Published:** 2026-08-11

**Authors:** Max Chapman, Muhammad Utomo, David J. Collins

**Affiliations:** ^1^ Department of Biomedical Engineering University of Melbourne Melbourne Victoria Australia; ^2^ Grame Clarke Institute University of Melbourne Parkville Victoria Australia

**Keywords:** 3D printing, alginate, biofabrication, microfluidics, wet‐spinning

## Abstract

We present a novel approach for the production of customizable microscale multimaterial flow cross‐sections. Here, a 3D‐printed array of microfluidic capillaries selectively maps numerous viscosity‐matched fluids to control the cross‐sectional geometry of a heterogeneous Poiseuille flow. Whereas previous work has demonstrated relatively simple axisymmetric flows, our approach is highly scalable to complex cross‐sectional geometries and large numbers of fluid inputs, and enables tailored cross‐sections without the limitation of radial symmetries, enabling the production of arbitrary fluidic ‘images’. We further design and implement an automated algorithm that generates a 3D model of an integrated flow extruder manifold tailored to the desired cross‐sectional geometry, which is then 3D printed. This design exploits laminar flow conditions to scale down cross‐sectional features, permitting an effective resolution amplification of two orders of magnitude from the minimum feature size of the 3D‐printed device. We demonstrate the spatial tuneability of the approach with a variety of periodic and asymmetric multimaterial flow geometries and for hydrogel microfibre production, in doing so utilizing up to 2347 individually defined fluidic pixels. With the ability to arbitrarily define flow cross‐sections with microscale precision, this approach has the potential to enable emerging applications requiring highly customized flows across optofluidic, biofabrication and lab‐on‐a‐chip devices.

## Graphical abstract

A 3D‐printed pixellated capillary array maps up to thousands of fluid inputs into arbitrary multimaterial flow cross‐sections. Automated manifold design and laminar scaling are used to amplify printing resolution orders of magnitude, generating microscale fluidic control from printed elements, enabling designed microflows for potential use in biofabrication, optofluidics, and lab‐on‐a‐chip applications.

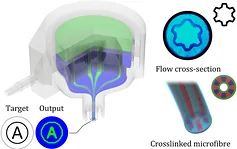

## 1 Introduction

Precise control of microscale fluid flows is vital for applications across biosensing [1, 2], drug discovery [3, 4], high‐throughput cell screening [5, 6], organ‐on‐a‐chip modelling [7, 8] and materials synthesis [9, 10, 11]. A key feature in many such applications is the ability to generate stable flows with multiple fluid inputs, aided by low Reynolds numbers intrinsic to microfluidic systems. For example, hydrodynamic flow focusing systems exploit fluid stresses at the boundaries of adjacent fluid streams for particle and cell focusing, mechanical probing and analysis of single cells [12, 13] and in droplet formation for microfluidic emulsions [14, 15]. Similarly, controlled fluidic concentration gradients are fundamental components of many microfluidic bioreactors [16, 17] and drug discovery platforms [18, 19]. Moreover, recent work has demonstrated the inclusion of liquid waveguides in optofluidic chips for biosensing [20, 21] and advanced photonics [22, 23, 24] applications.

Such microfluidic devices are predominantly fabricated using planar architectures, generating 2D networks of rectangular‐section channels. These geometries offer precise control of fluid composition and flow velocity in the horizontal plane, but offer limited control in the out‐of‐plane (vertical) axis. Developments in the field of flow lithography have enabled the formation of two or more fluid layers in the out‐of‐plane axis via the implementation of multilayered PDMS channel networks [25]. However, the requirements for precise alignment between adjacent layers of the PDMS chip bring a practical limit to the achievable complexity of such flow substructures. Similarly, inertial [26] and non‐inertial [27] flow sculpting techniques permit the excitation of specific nonplanar flow geometries using geometric features in microfluidic channels that perturb flow in a controlled fashion. Despite recent efforts to expand the flexibility of this approach using AI‐based design of flow sculpting features [28], these approaches limited control of flow geometry due to the indirect relationship between the channel and flow geometries.

An alternative approach, glass capillary microfluidics, involves the coaxial assembly of glass capillaries of different sizes to form concentric layered flow profiles. This, however, limits the ability to sculpt non‐axisymmetric concentration fields for control of chemical reactions [29], create designed particles in barcoding applications [30, 31], and locally tailor asymmetric fluid properties for optical beam shaping [24]. Accordingly, direct control of the complete two‐dimensional flow cross‐section would be of substantial benefit for these applications, but remains challenging.

To create more complex flows, recent efforts have realised a degree of substructural control in the azimuthal axis of sheath‐core flows for advanced microfluidic wet spinning [9], stop‐flow particle fabrication [32, 33] or 3D‐printing [34] applications. Cross‐sectional substructures in the former case are achieved by manual assembly of several glass capillaries into bundles, allowing fluid flow to be segmented for multiple input fluids [9]. Meanwhile, customized 3D‐printed nozzles enable the extrusion of simple radially symmetric multicompartmental geometries into square [32, 33] or round [34] channels. Nevertheless, these approaches are limited to relatively simple cross‐sectional geometries due to practical limitations in assembling glass capillary bundles and geometric constraints in the design of concentric layered nozzle geometries.

In this work, we present a highly scalable workflow for generating multimaterial microfluidic flows with arbitrary cross‐sectional compositions. Our multimaterial extrusion technique combines two enabling features, namely a 2D array of microfluidic capillaries arranged orthogonally to the direction of flow, and a series of layered planar fluidic manifolds, each of which can be arbitrarily connected to any number of interconnecting capillary vias. This arrangement offers a generalized architecture for the infusion of numerous fluids with individually addressable fluid “pixels,” which here has been optimized for fabrication using low‐cost 3D printing. Alongside advances in geometric complexity, demonstrating between 91 and 2347 fluidic pixels in our customizable fluidic arrays, we also report an orders‐of‐magnitude reduction in feature size between the 3D‐printed array geometry and the resultant fluid pixels via laminar flow focusing. The accessibility and scalability of our technique is further supported by a Python workflow that directly interfaces with CAD software to automate the design of capillary array devices, including distortion compensation to account for the development of fully developed flow along the fluidic manifold. As this work enables the arbitrary design of cross‐sectional flows in a way that is unparalleled by previous multimaterial flow techniques, this work is highly relevant for emerging applications in advanced microfluidics, optofluidics, and high‐throughput microfabrication.

## 2 System Principles

### 2.1 Pixellated Multimaterial Flow

The pixellated multimaterial flow approach incorporates a scalable 2D array of microfluidic capillaries to extrude two or more viscosity‐matched fluids into a tubular outlet (Figure 1a). This results in the ability to arbitrarily design the fluidic cross‐section comprising two or more fluids. Here, each capillary is mapped to one of the available fluid phases and services an equal volumetric flow rate, resulting in a multicompartmental flow in which the flow exiting each capillary forms a “fluidic pixel.” Due to the controlled capillary flow rates, the pixellated flow exhibits a uniform velocity distribution immediately downstream of the capillary array; this velocity profile evolves into hydrodynamically‐focussed laminar Poiseuille flow prior to exiting the outlet nozzle.

**FIGURE 1:**
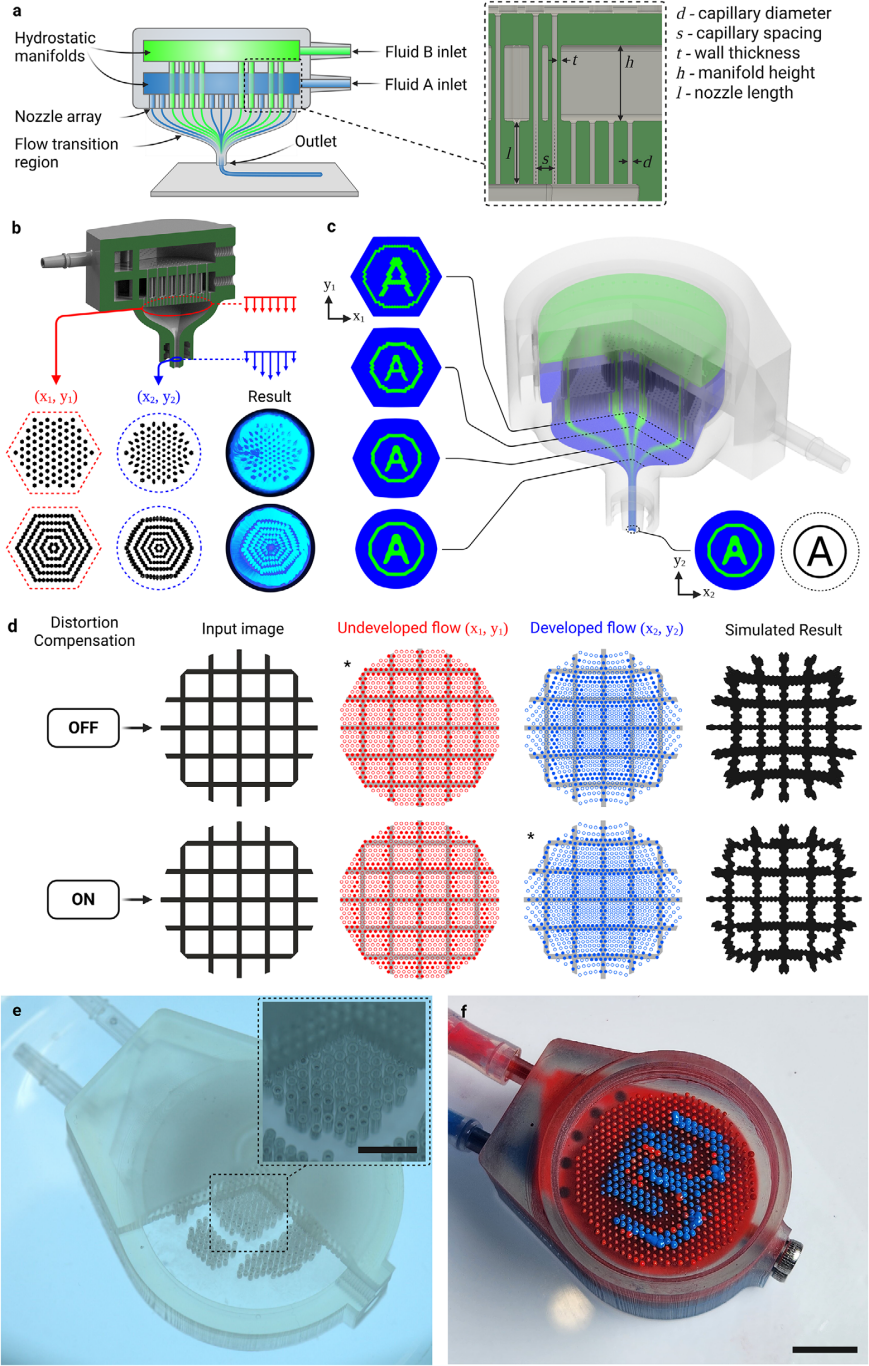
Pixellated multimaterial flow extrusion principles. (a) Schematic illustration of a two‐fluid extrusion system. (b) Experimental validation of the numerically obtained distortion functions for hexagonal checkerboard (top) and ring (bottom) calibration arrays. (c) CFD simulation showing the evolution of the distorted flow over the length of the flow transition region. (d) Schematic representation of distortion compensation algorithm, showing pixel locations in coordinates corresponding to undeveloped (x_1_, y_1_) and fully‐developed (x_2_, y_2_) flow. Pixel assignments (denoted by ^*^) are sampled in either (x_1_, y_1_) or (x_2_, y_2_) depending on whether distortion compensation is applied. (e) Digital photograph of example extruder device with cutaway section. (inset) close‐up view of capillary features. Capillary diameter is 400 µm and center‐to‐center spacing of adjacent capillaries is 1.0 mm. (f) Example device with outlet channel removed and partially perfused with acrylic paint. Scale bars = 5 mm (e), 10 mm (f). The CBML Logo design pictured here is reproduced with permission from the Collins BioMicrosystems Laboratory.

Included in the capillary array device are a series of vertically‐stacked planar manifolds, each of which services one fluid input. Fluid‐pixel assignments are achieved via internal capillary structures that project upwards from the capillary array to the corresponding manifold, protruding through intermediate manifold regions. Each manifold has critical dimensions much larger than the capillary diameter, such that flow velocities are comparatively small, resulting in quasi‐hydrostatic conditions across capillary inlet locations. Accordingly, this arrangement results in equivalent flow rates across capillaries assigned to a particular fluid. Finally, the fluid inputs are externally controlled via syringe pumps, with volumetric flow rates in proportion to the number of connected capillaries such that each capillary services an equal flow rate independent of fluid assignment and array position.

A convenient feature of this approach is the ability to arbitrarily reduce the scale of the extruded multimaterial flow via laminar flow‐focusing. Here, cross‐sectional flow features are first assembled at the millimetre scale prior to entering the converging outlet channel. Operating in the low Reynold's number regime, these cross‐sectional features can thus be retained while undergoing scalable increases in cross‐sectional flow pixel resolution, representing an orders‐of‐magnitude increase across the demonstrated devices in this work. In contrast to previous work using converging flows for feature size reduction [35], the converging channel in our system is much larger than the size of assembled fluid pixels. This feature theoretically allows for more dramatic resolution amplification using our method.

### 2.2 Distortion Modeling and Compensation

The fluid flow cross‐section at the outlet of the capillary tube vias is not identical to that found at the converged outlet, where the transition from pixellated/uniform to fully developed flow along the converging flow geometry needs to be accounted for. Accordingly, two factors contribute to distortion of the cross‐sectional geometry between the capillary array and the device outlet. Firstly, a radial distortion component exists due to the transition from the undeveloped flow at the start of the transition region to fully‐developed Poiseuille flow at the outlet nozzle. After entering the flow transition region, fluid near the center accelerates more rapidly than fluid near the edges as the flow profile evolves naturally into a parabolic velocity distribution. As each fluid “pixel” is fed with the same volumetric flow rate, pixels near the center of the cross section become increasingly smaller than surrounding pixels as flow evolves to its steady‐state Poiseuille configuration near the nozzle outlet.

Secondly, whereas the nozzle outlet utilizes a circular cross‐section, the capillary tube layouts utilized invariably result in regular n‐polygonal truncated shapes when comprising finite numbers of capillaries. Here, the capillary arrangement can be simplified via the utilization of capillary arrays whose unit‐cell composition mirrors that of the overall extent (e.g., hexagonal arrays resulting in a hexagonal shape). As such, where we demonstrate the use of both hexagonal and circular‐cropped capillary arrays in our experiments, a second distortion component may exist due to shape transition between the capillary array boundaries and the device outlet. In this case, the flow cross‐section evolves from a hexagon to a circle over the length of the flow transition region, which takes the form of a 3D loft geometry. Figure 1b shows the combined effect of both distortion components for a hexagonal array using checkerboard and concentric ring calibration geometries.

Using the COMSOL Multiphysics laminar flow module, the flow evolution over the transition region was numerically simulated. Figure 1c shows the simulated evolution of an example geometry over the transition region for a hexagonal array design. From these results, we generated normalized transformation functions characterizing the transition from array space (denoted (x_1_, y_1_), corresponding to undeveloped flow) to outlet space ((x_2_, y_2_), corresponding to fully developed Poiseuille flow) for both cropped‐circular and hexagonal capillary array configurations. Figure S5 shows the resultant transformation functions for both hexagonal and circular cropped distortion models; polynomial functions for both regimes are provided in Section S5.1. Distortion compensation is then achieved by sampling the desired geometry in outlet space using the predicted x_2_, y_2_ pixel locations, mapping these streamlines back to those of the array space (x_1_, y_1_). Figure 1d shows the effect of distortion across the flow geometry without compensation, where mapping the desired image directly to the capillary tube layer results in significant magnification of pixels at the nozzle edges, contraction of pixels in the middle of the flow, and distortion of flow features that span across the flow cross‐section in non‐radial directions. Applying our compensation approach based on these transformation functions, however, allows us to back‐calculate the capillary tube layout that results in the desired flow cross‐section at the post‐transition, steady‐state Poiseuille flow condition at the nozzle exit.

### 2.3 Automated 3D Design of Capillary Array Devices

To facilitate the rapid iteration of flow extruder prototypes and enable the design of flow cross‐sections that would be prohibitively complex for manual segmentation, we developed a custom automated Python workflow that interfaces with the Fusion 360 Application Programming Interface (API). Firstly, an image sampling script converts an input bitmap image to a series of normalized capillary coordinates for each fluid phase according to the user's desired parameters (array pixel resolution, array shape, and number of fluid inputs). Secondly, we have designed a parametric CAD model of a generic extruder device (containing internal manifold structures but no capillary features). With the template model open in Fusion 360, running our Python plugin initially resizes the template geometry according to the array size (*n*), capillary dimensions (*s*, *d*, *t*), and number of fluid inputs (*p*), then builds capillary features according to the calculated pixel coordinates, resulting in an STL file that can then be 3D printed. Figure 1e,f shows example 3D‐printed capillary array devices with design parameters *s*  = 1 mm, *d*  = 0.40 mm, *t*  = 0.20mm, exhibiting the internal capillary geometry via a section cut (Figure 1e) and fluid‐pixel assignments with the outlet channel removed (Figure 1f). Figure S2 summarizes the capillary array design process in flowchart form.

## 3 Results and Discussion

### 3.1 Pressure Distribution Analysis

Critical to the function of the capillary array device is the concept that each capillary in the array services a similar volumetric flow rate, yielding a fluid cross‐section relatively free of distortion relative to the target image. This assumption is valid for a given range of the design parameters *n*,  *h*,  *d*,  *s*,  *t*, with the goal of limiting the pressure variation within each of the manifold regions, where *n* is the total number of capillaries in the array, *h* is the manifold height, *d* the internal diameter of each capillary, *s* the capillary spacing and *t* the capillary wall thickness (as noted in Figure 1a). To characterize the geometric constraints arising from this assumption, we introduce the following constraint over the entire fluid volume

(1) $$\min\left[\Delta P\left(\mathrm{capi}\mathrm{llary}\;\mathrm{array}\right)]\gg \max[\Delta P\left(\mathrm{mani}\mathrm{fold}\right)\right].$$

Firstly, local fluctuations in manifold pressure between neighboring capillaries are kept small by selecting *h*,  *d*,  *s*,  *t* such that the pressure drop across the length of a capillary, Δ*P_c_* (Equation S5), is much greater than the pressure difference between neighboring capillaries, Δ*P_UC_* (Equation S3), due to flow moving horizontally within the manifold. This ensures that horizontal pressure gradients within the manifold remain small and thus neighboring capillaries receive approximately equal input pressure, irrespective of symmetry in the assignment of fluid pixels.

It should be noted that such small gradients may still yield a large pressure differential if maintained over a sufficiently large distance. Hence, in addition to flow properties at the unit cell scale, we must also consider field properties due to the arrangement of capillaries within each manifold. Due to the single fluid input on one side of each manifold, there exist both radial and horizontal pressure gradients within the manifold regions (Figure 2b). However, with the appropriate use of a radial diffuser, the radial component dominates the pressure field (as exhibited in Figure 2d, where the radial pressure gradient is an order of magnitude greater than the horizontal one). Here, the radial diffuser feature improves the axial symmetry of the pressure field within the manifold region; more detail on the inclusion of specific design features is included in Figure S4. Accordingly, in our mathematical treatment we assume an axisymmetric pressure field, as this permits evaluation of the worst‐case flow occlusion conditions for a given set of array parameters. To evaluate the inequality in Equation (1) and validate pressure uniformity, we therefore examine the worst‐case region as a central cluster of *k* pixels in the lowest manifold, in a capillary array design representing maximum occlusion from surrounding capillary structures (Figure 2b). In the general case, Equation (1) is characterized by the pressure values at points *P*_1_, *P*_2_ and *P*_3_ as depicted in Figure 2a, with

(2) $$\frac{\Delta P_{12}}{\Delta P_{23}}<0.05\;$$

**FIGURE 2:**
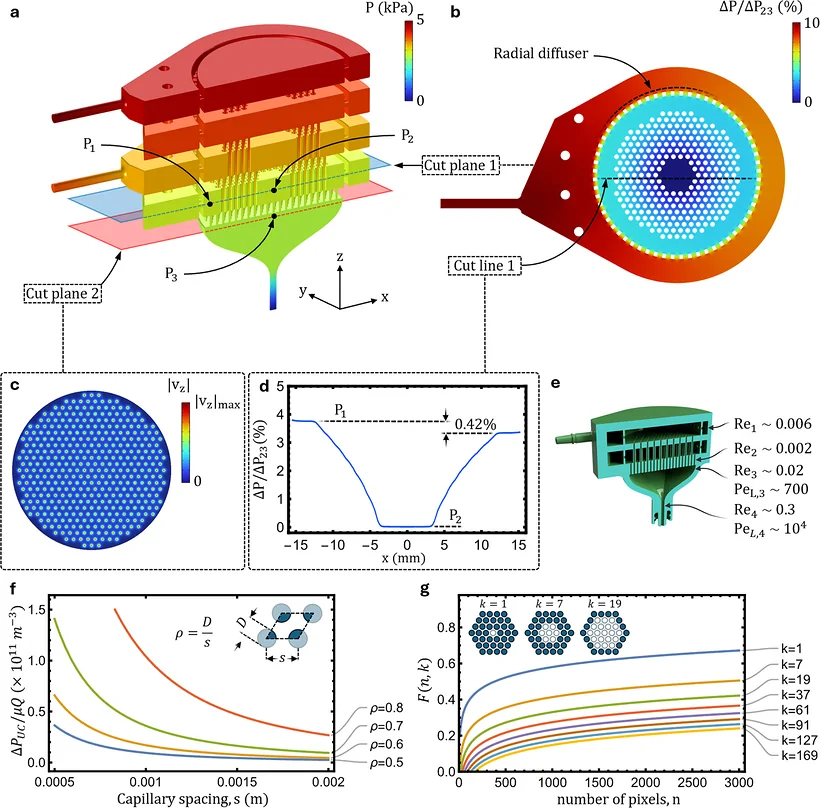
Internal pressure field modelling. (a) Section view showing pressure distribution within example four‐fluid extruder device. Cut plane 1 (blue) depicts the surface along which pressure is plotted in (b), and is located along the midsection of the lowermost manifold. Cut plane 2 (red) depicts the surface along which z‐velocity is plotted in (c), and is located one capillary diameter (0.5 mm) below the capillary outlets. (b) Manifold pressure distribution along cut plane 1, normalized to capillary pressure Δ*P*_23_. Cut line 1 depicts the axis along which pressure is plotted in (d). (c) Surface plot showing z‐axis velocity distribution immediately below the capillary array. (d) Pressure distribution along cut line 1, normalised to the minimum capillary pressure Δ*P*_23_. (e) Approximate Reynold's and Peclet numbers for critical regions in an example dual‐fluid capillary array device using μ  =  1.412 Pa s (glycerol, 20°C) for the reference viscosity, *Q*  =  1 mL/min and a diffusion coefficient of 10^−10^ m^2^/s (approximate for aqueous dye in pure glycerol [36]). Critical regions include manifolds (*Re*_1_), capillaries (*Re*_2_) and the start (*Re*_3_,*Pe*_*L*,3_) and end (*Re*_4_,*Pe*_*L*,4_) of the flow transition region. (f) Normalised unit cell pressure drop Δ*P_UC_*/μ*Q* as a function of capillary spacing *s* and diametral ratio ρ, with a fixed manifold height of *h*  =  5 mm. (g) Pressure drop scaling factor *F* (*n*, *k*) = Δ*P*_12_/Δ*P_UC_* as a function of the total number of fluid pixels, *n*, assuming maximal occlusion in the lowermost manifold.

Here, we apply the Brinkman Equation [37] (Equation S1) to estimate the fluid resistance of a confined field of micropillars within the manifold region, using the relationship derived by Tamayol and Bahrami [38] (Equation S2) to calculate the permeability, *K*, of a hexagonally‐staggered array of micropillars. This treatment estimates the fluid resistance of a unit cell of pillars within the manifold, Δ*P_UC_*/μ*Q* (Equation S3), the results of which are shown in Figure 2f for a given pillar spacing, *s*, and relative pillar diameter, ρ, where ρ  =  *D*/*s*, for a fixed manifold height of *h*  =  5 *mm* (typical in our experiments). To characterise the influence of geometric arrangement of capillaries within the manifold volume, we then apply a scaling factor, *F* (*n*, *k*) =  Δ*P*_12_/Δ*P_UC_* (Equation S7), which estimates the pressure drop due to radial flow through an annular field of *n* − *k* unit cells around a central cluster of *k* capillaries (Figure 2g, Figure S1a). Here the worst‐case pressure gradient in the manifold (characterized by large $\frac{\Delta P_{12}}{\Delta P_{23}}$) occurs at a point such that 1 < *k* < *n*, plotted in Figure S1b. Finally, the minimum capillary pressure drop can be estimated using the Hagen–Poiseuille Equation (Equation S5), allowing evaluation of Equation (2).

### 3.2 Cross‐Sectional Image Analysis

To evaluate the capacity of our system to accurately reproduce a range of cross‐sectional geometries, we performed several tests with a range of sample images and array sizes using in‐flow imaging of dyed glycerol flows. The geometric accuracy of extruded image features can be characterised via several qualities, including the circularity of round features, straightness of straight lines, edge smoothness, minimum feature size, magnitude of horizontal distortion and the presence of image defects.

Figure 3 examines the generated flow cross sections for a range of input images, examining the relationship between pixel number, image complexity and resulting flow. Larger pixel numbers enable a reduction in minimum feature size, and improve the accuracy of reproduction of thin or sharp features due to reduced discretization error. A secondary effect of increased sampling resolution is to improve the reproduction accuracy of simple geometric features, e.g., circles and straight lines, via the improved efficacy of the distortion compensation algorithm. Due to the discrete nature of the pixel array, there is a finite threshold in the distance between a pixel's location in array space (x_1_, y_1_) and outlet space (x_2_, y_2_) under which distortion compensation will not take effect. Increasing the sampling resolution lowers this threshold by reducing the distance between adjacent pixels in (x_2_, y_2_) (noting that, due to the irregularity of the distorted array, this distance is spatially variable) and increasing the likelihood that a pixel's assignment will change during the distortion compensation process. To quantify the accuracy of reproduction of target images, we performed Intersection over Union (IoU) analysis of the images shown in Figure 3a, finding a general trend of increasing IoU (as defined in Equation S3) with array size, *n*. Further detail on the analysis methods and results is provided in Section S2.

**FIGURE 3:**
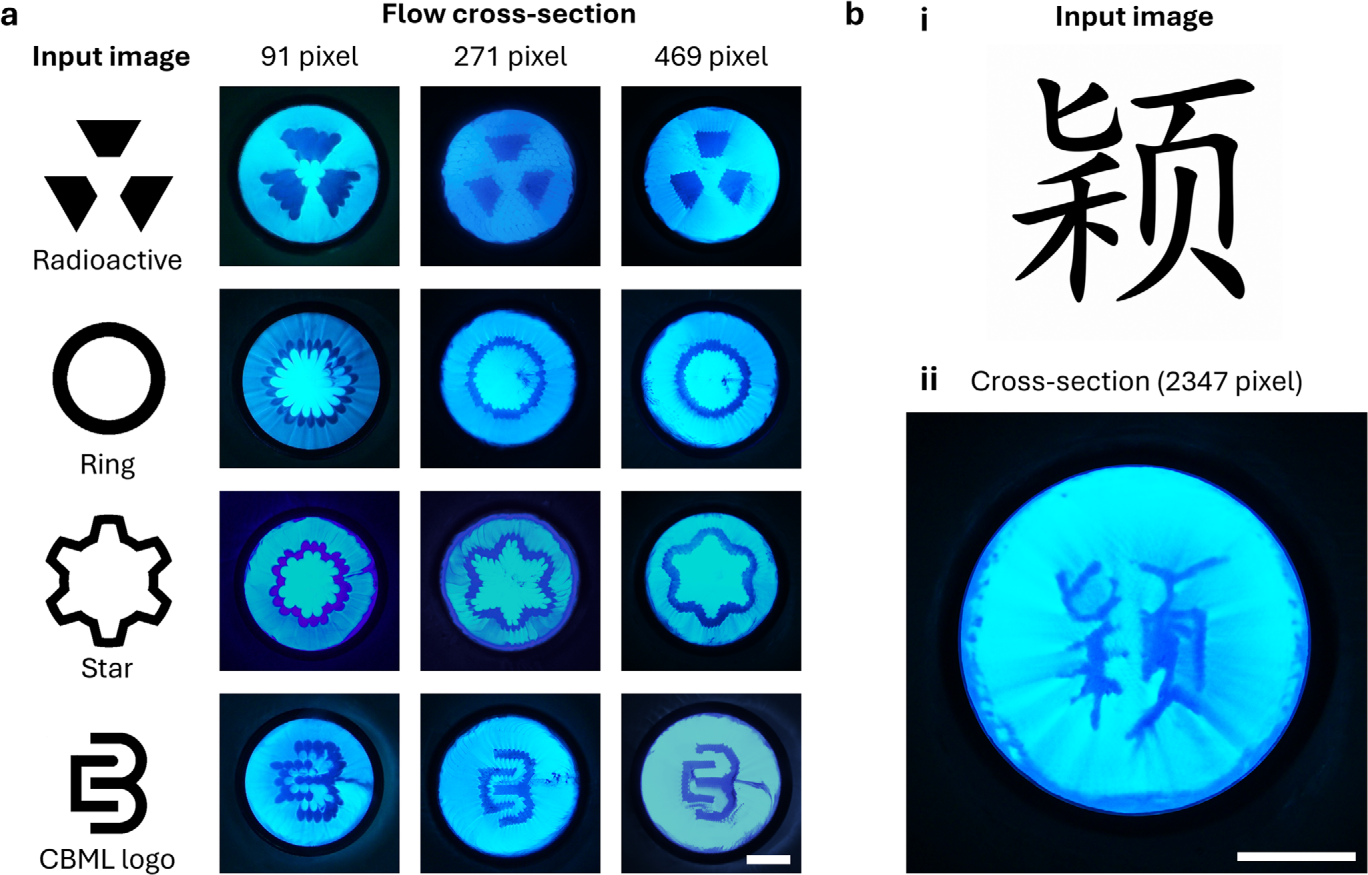
In‐flow imaging of example cross sections. All geometries were processed with distortion compensation applied and extruded using undyed and blue‐dyed glycerol as the two fluid inputs. (a) Parameter sweep showing Radioactive, Ring, Star, and CBML Logo target geometries, each sampled with pixel numbers *n*  =  91,  271, and 469. The CBML Logo design is reproduced with permission from the Collins BioMicrosystems Laboratory. (b) Simplified Chinese character “yǐng” sampled with an array size of 2347 pixels. Scale bars = 500 µm.

To highlight the influence of pixel resolution on specific image features, we note that the triangular “radioactive” design (Figure 3a, first row) exhibits little improvement in reproduction accuracy with increasing resolution, where the radial edges of the image are parallel to the hexagonal pixel array, while the “ring” feature (second row) displays a marked improvement in circularity due to reduced discretization error and the increased influence of distortion compensation (noting that for the 91‐pixel “ring” design, distortion compensation yielded no change to the pixel assignments due to insufficient pixel density). Further, the “star” design displays improved edge smoothness due to the smaller feature size, while the “CBML logo” design exhibits increasing accuracy in the reproduction of rectilinear edges as discretization error is minimized.

Uniquely, our approach enables the ability to generate designed cross‐sectional flow features with no radial or azimuthal symmetry. While some recent work has extended capillary microfluidics beyond its traditional limitation to purely axisymmetric flows, flow cross‐sections were still limited to those with linear or radial symmetry, i.e., features that appear identical when mirrored about a centerline or at multiple rotated orientations [9, 34]. Figure 3b,c accordingly demonstrates flow features devoid of geometric symmetry, allowing the reproduction of irregular or organic shapes here with feature sizes of ∼30 µm (at the nozzle outlet).

Image defects were occasionally encountered throughout testing, usually appearing as a dark spot or streak in an unexpected region. These are likely caused by physical defects in 3D‐printed capillary array devices, where a crack in the capillary wall allows one of the fluid inputs to leak into an adjoining manifold and contaminate the adjacent fluid. Such defects are often the result of small air bubbles intersecting with capillary walls during the 3D printing process, causing weak points that rupture under operating pressure, and occur more frequently when testing large array sizes (*n* > 400), suggesting that their occurrence is statistical in nature. The use of specialised 3D printing technologies with improved resin processing may mitigate this issue in future work.

### 3.3 Multicompartmental Microfibres

Whereas Figure 3 demonstrates the ability to generate designed cross‐sectional flow patterns, here we examine the generation of designed extrusions, in which these patterns are retained in a solid gel that cures immediately after nozzle ejection. Combining our approach with wet spinning approaches [10] accordingly enables the synthesis of soft microfibres with similar flexibility in the cross‐sectional geometry, including for the generation of multimaterial and hollow substructures. We demonstrate this utilizing a sodium alginate solution, a crosslinkable hydrogel with good biocompatibility and widespread use in bioinks [39, 40]. Here, the alginate solution is extruded from the nozzle into a calcium chloride bath, resulting in rapid cross‐linking and gel formation, thus maintaining the designed cross‐section present at the nozzle exit. For solid microfibres (Figure 4a–e), pigmentation was applied to the parent hydrogel solution to visualize multicompartmental substructures. Here we highlight the significant cross‐sectional resolution of microfibres produced using our approach (e.g., 859 microfibre “pixels” in Figure 4b), as well as the inclusion of four multicoloured fluid inputs (Figure 4c–e), both of which designs exceed the cross‐section complexity available to previous extrusion approaches [9, 34].

**FIGURE 4:**
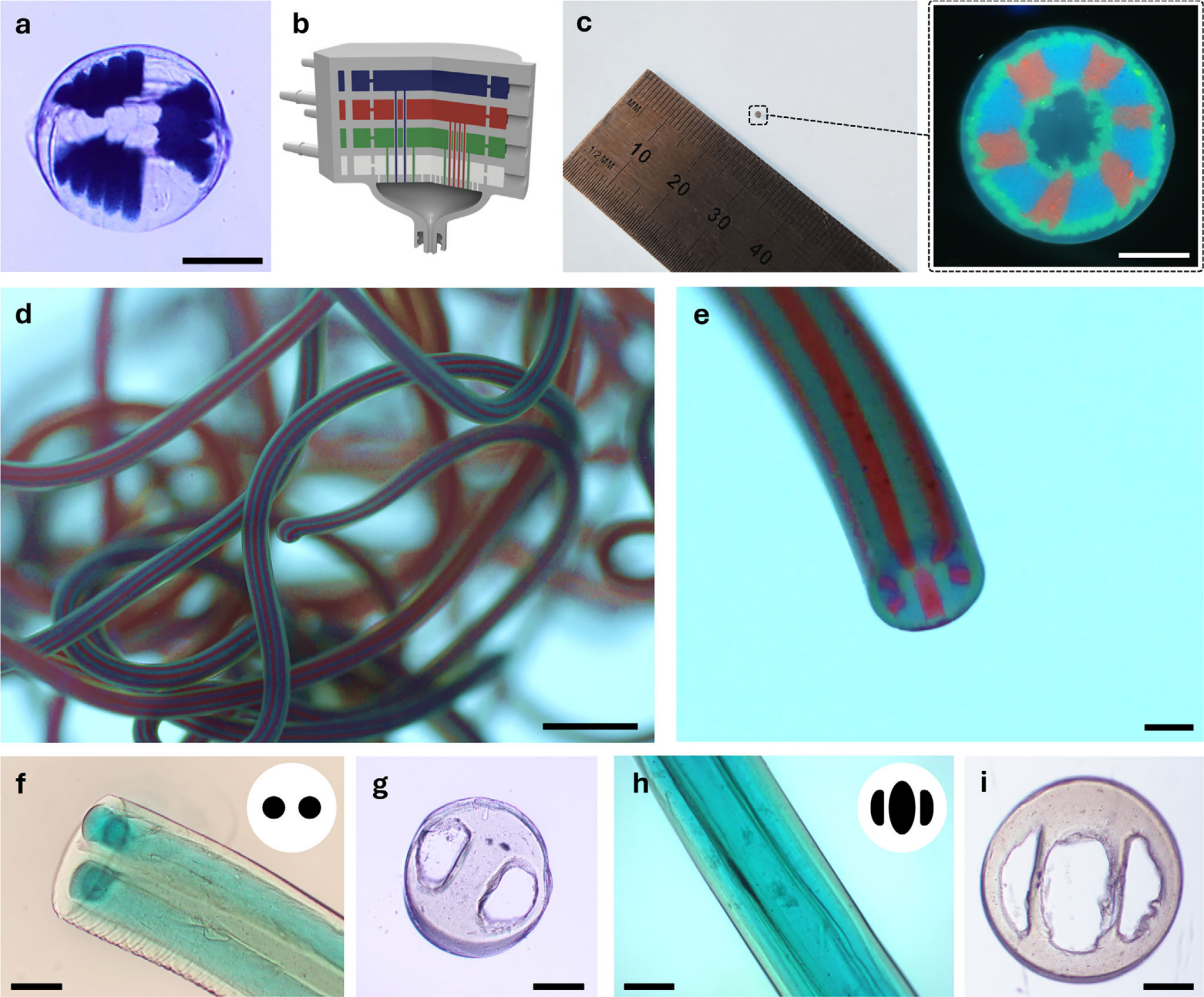
Crosslinked alginate extrusions. (a–f) Solid filaments with multicoloured substructure. (a) 91‐pixel “radioactive” filament slice in blue/clear. (b) Cutaway view of four‐input device used to produce extrusions in (c–e). (c) Macro photograph of four‐colour compound geometry in red/blue/green/clear with millimetre ruler for scale. Inset: darkfield image of filament slice. (d, e) Macro photographs of continuous four‐colour compound microfibre. (f–i) Perfusable capillary extrusions. (f) Dual‐lumen microfibre primed with blue‐dyed PVA solution. (g) Dual‐lumen microfibre slice. (h) Tri‐lumen perfusable microfibre. (i) Tri‐lumen microfibre slice. Scale bars: 500 µm (a, c, e‐i); 5 mm (d).

Utilizing a combination of inputs, including those that are non‐crosslinkable, further enables the formation of material cross‐sections comprising hollow elements/voids. In Figure 4, hollow microfibres were fabricated utilizing a viscosity‐matched PVA template solution as a sacrificial fluid material, co‐flowing with the alginate solution from the final manifold stage (Figure 3f–i). After extrusion and subsequent crosslinking, the PVA solution was removed via aqueous diffusion through the hydrogel walls [41], after which individual lumens within the microfibre can be perfused (Video S1). This approach enables the production of perfusable channels with complex cross‐sections that are not achievable with planar fabrication techniques such as soft lithography, enabling functional structures such as the variable‐aperture tri‐lumen structure shown in Figure 3h,i.

### 3.4 Discussion and Future Perspectives

Our capillary array design enables the convenient and stable production of multimaterial fluid flows, with markedly increased control of the two‐dimensional flow cross‐section over previous approaches. In characterising its operational envelope as a function of fluid viscosity, flow rate, and the geometric constraints relating to device fabrication, we demonstrate its scalable application to different fluids across pixel number, designed cross‐sectional complexity, and number of discrete fluidic inputs. Further, we utilize a flow‐focusing fluidic manifold prior to the nozzle exit, which enables the generation of fluidic feature sizes considerably smaller than the minimum feature size of the 3D‐printed fluid manifold. Lastly, we have demonstrated that, excluding the presence of occasional physical defects likely due to irregularities in the 3D printing process, capillary array devices can be repeatedly reused without issues provided they are primed correctly and rinsed with DI water after use. This includes both the extrusion of pixellated liquid flows and microfluidic wet spinning of multicompartmental microfibres.

A key novelty of this approach is the parameterisation of the capillary array design that, combined with its 3D architecture, enables very large numbers of pixels or fluid inputs within an automated design framework, where a designed cross‐section can be used to rapidly generate a capillary flow array. Nevertheless, practical considerations prevent the performance of very large flow geometries, where large *n*,  *p* necessitate greater device dimensions, with impacts on flow stability, internal pressures, and image defects if other design parameters (e.g., printing resolution) are kept constant. In our experiments, limitations in the feature size achievable using consumer‐grade 3D printing constrain the capillary spacing to the order of 1 mm or larger, meaning that arrays with > 1000 pixels necessitate capillary array dimensions of the order of 5 cm. Despite operating in the low Reynold's number range (Re < 1), the stability of the flow in the flow transition region is less consistent with large devices when using viscosity‐matched dissimilar fluids. Here, a low‐Re transition to turbulence is enhanced by viscosity mismatches between fluids, which, due to the non‐Newtonian nature of hydrogel solutions, is unavoidable given the variable shear rates occurring within the capillary array device (see Figure S6). Accordingly, while in this work we demonstrate the ability to generate microscale flow features utilizing consumer‐grade 3D‐printing, future explorations may investigate the application of specialised high‐resolution 3D‐printing processes to further reduce Re, such as projection micro stereolithography (PµSL) [42, 43] or two‐photon lithography (2PP) [44] to achieve increased image complexity beyond thousands of pixels, while still minimizing image distortions.

Such stable multimaterial flows with precisely controlled substructure have intrinsic utility across microfluidics and optofluidics. This includes applications in optofluidic waveguides and environmental sensors, where the ability to manipulate fluid properties (for example, concentration gradients or refractive indices) across the entire two‐dimensional flow cross section is desired, and in specialised microfluidics applications such as micromixing [45]. Moreover, designed multicompartmental microfibres have the potential to realize specialised mechanical or optical properties in filament metamaterials [34], photonic crystal fibres [46] or for hydrogel scaffolds in tissue engineering. Our approach is further well suited for integration with existing suspension‐bath 3D bioprinting techniques, in which bioink is extruded from a syringe needle into a viscous fluid bath [47]. The inclusion of a viscosity‐matched template solution also enables the inclusion of complex perfusable channel geometries within wet‐spun microfibres (e.g., Figure 4g–j), where engineered internal structures with high surface area to volume ratios enable controlled heat‐exchange or ion‐exchange experiments. We envision that our flow design and filament manufacturing approach will enable a new range of mechanically functional microfluidic channel designs for lab‐on‐a‐chip cell analysis, and biologically relevant lumen‐based modeling.

## 4 Conclusions

In this work, we present a pixel capillary array‐based approach for generating multimaterial fluid flows, with the unique ability to generate designed flow cross‐sections. These capabilities are supported by a highly parameterised design and automated workflow, enabling scalability over a range of array dimensions, length scales and flow geometries. We accordingly demonstrate the ability to generate designed flows from thousands of individually assigned capillary‐based fluid pixels. Importantly, this is not limited to pairs of fluid inputs, where the number of fluid inputs is scalable as well; here we generate fluid flows combining up to four separate fluidic inputs. By cross‐linking the ejected flow, we further establish the ability to create extruded multimaterial solids and hollow microfibres, with a designed number and arrangement of embedded lumens. As a result, our array system offers significant benefits in a range of microfluidic, optofluidic, biofabrication and materials science applications. While this work demonstrates the ability to generate designed flows from consumer‐grade 3D printed geometries, the implementation of yet higher‐resolution 3D printing systems has the potential to further enable the inclusion of yet higher pixel numbers, enabling further enhancements in cross‐sectional resolution.

## 5 Methods

### 5.1 Device Fabrication

Capillary array devices were fabricated using an AnyCubic Photon Ultra benchtop DLP SLA 3D printer using AnyCubic Clear standard 3D printer resin. Devices were printed in the vertical orientation such that internal capillary axes were orthogonal to the build plate, ensuring minimal shear and bending stresses were applied to delicate capillary structures during the printing process. Chitubox Basic V2.3.1 was used to postprocess STL files for printing. For all devices used in our experiments, a layer height of 100 µm and a layer exposure time of 3.0 s were used. A detailed investigation of the effect of surface roughness on device performance was not included in the scope of this work; the effects of surface irregularities were instead limited by ensuring the ratio $\frac{\eta }{d}\leq 0.1$, where η is the 3D printing layer height and *d* is the critical spacing between neighbouring capillaries. External support structures were manually applied during the slicing process to support the integrated tube fittings at the fluid inlets; otherwise, no other support structures were used. After printing, parts were removed from the build plate and manually washed twice using pure isopropyl alcohol, with visual inspection of internal manifold structures after each wash to ensure adequate clearing of uncured resin. Parts were then dried using compressed air and cured at room temperature for two minutes using a Formlabs Form Cure station.

### 5.2 In‐Flow Imaging

An end‐on imaging approach was implemented to evaluate the cross‐sectional geometry of fluid flows. Here, dual‐fluid extrusion systems were set up using pure glycerol and a 0.1% w/w mixture of blue food dye in glycerol. Before priming with glycerol, capillary array devices were first wetted with isopropyl alcohol prior to perfusion with glycerol, as this improved wetting and facilitated priming with minimal air bubbles. The output of the capillary array device was then connected to a 14G, 1.25″ syringe needle and the needle tip suspended approximately 0.5 mm above the base of a petri dish (see Figure S3). The capillary array and petri dish assembly were installed into a benchtop inverted microscope (Olympus IX73) and imaged from underneath using top‐down lighting, such that the light transmits through the body of the capillary array device and down the length of the needle. Typical in‐flow imaging experiments were performed with a combined flow rate of the order of 1–5 mL/min, as this range enabled device priming over timescales of the order of a minute, while keeping internal hydrodynamic pressures low enough to avoid rupture of device walls. Early glycerol perfusion experiments at flow rates greater than 10 mL/min showed observable bulging of manifold walls and occasional rupture of internal capillary structures. During normal operation, the extruded flow hits the base of the petri dish and spreads radially. Due to the comparatively large needle length in comparison to the 0.5 mm fluid thickness below it, the resultant image is dominated by light absorption within the needle and only minimal ghosting can be seen due to the radial spread of fluid below the needle tip. Video S2 shows the real‐time evolution of an example glycerol flow geometry.

### 5.3 Alginate Microfibre Gelation

Alginate microfibres are produced using a 5% w/w sodium alginate ((C_6_H_7_NaO_6_)_n_) solution (W201502, Sigma‐Aldrich) for one or more of the fluid inputs and immersing a 14G or 18G outlet needle in a 10% w/v bath of aqueous calcium chloride (CaCl) solution (223506, Sigma‐Aldrich). For such experiments, combined flow rates in the order of 10 mL/min or higher are generally required to prevent the formation of globular droplets at the end of the outlet needle. Under these conditions, the exterior of the hydrogel filament skins immediately upon exiting the device outlet due to ion‐exchange crosslinking, after which the filament is left immersed in the calcium chloride solution for 5 min to ensure complete crosslinking of its internal structure. Where multicoloured filaments were used to visualize substructural features, dry artist's pigments (Langridge Colours, Australia) or standard acrylic paint was added to alginate parent solutions at 2% w/w concentrations. After use, capillary array devices were rinsed thoroughly with DI water to prevent blockages caused by dried sodium alginate or residual pigment remaining in the internal capillary channels.

### 5.4 Viscosity Matching of PVA Template Solution

Where perfusable filaments were generated, the 5.0% w/w sodium alginate solution was infused alongside a viscosity‐matched aqueous polyvinyl alcohol (PVA) template solution. The PVA solution was then allowed to diffuse naturally through the microfibre walls by soaking the microfibre in deionized water for approximately 30 minutes after the alginate structures were sufficiently crosslinked.

The viscosity‐matched PVA solution was prepared by mixing 14.7% w/w hydrolysed PVA (P8136, Sigma‐Aldrich) with boiling deionized water and mixing on a reciprocating shaker for 24 h. Prior to preparing the PVA template solution, a numerical simulation was performed for a typical flow extruder device using COMSOL Multiphysics laminar flow module, showing shear rates in the range 100–500 s^−1^ over the area of interest within the hydrodynamic focusing region. Areas with shear rates below 100 s^−1^ were ignored as the flow profile was approximately uniform in these regions. Rheological tests were then performed (Anton Paar MCR 702 TwinDrive Rheometer) to determine the shear‐viscosity curve of the experimental 5% alginate solution, showing significant shear‐thinning behaviour with dynamic viscosity ranging from approximately 180–215 mPa s over the 100–500 s^−1^ shear rate region of interest (Figure S6a). Rheological tests were also performed to determine the concentration‐viscosity curve of the aqueous PVA solutions over the range 13%–19% w/w PVA in 1% increments (Figure S6b). In order to achieve an approximate viscosity match between the 5% w/w alginate and PVA template solutions, the average dynamic viscosity μ_*avg*_ =  194 mPa s over the 100–500 s^−1^ shear rate region of interest was assumed as the target viscosity for the PVA buffer solution, corresponding to an aqueous PVA concentration of 14.7% w/w.

Specific efforts to match the density of the coflowing fluids were omitted from our investigation; however, we highlight that the alginate‐PVA fluid combinations used in our experiments inherently result in only small density mismatches of the order of 1–2% (while in the case of glycerol‐glycerol + food dye experiments, there was no measurable difference in density).

In the case of the alginate‐PVA fluid combination, the strength of the density disparity is characterized by the Atwood number,

$$A=\frac{\rho_{h}-\rho_{l}}{\rho_{h}+\rho_{l}}.$$

Taking ρ_*h*_ ≈ 1.03 (14.7% PVA) and $\rho_{l}∼1.01$ (5.0% alginate) gives the approximate result A∼0.01. At this point, we consider the stability of a fluid interface within our system with periodic perturbations on the order of the capillary spacing, λ  =  *d*  =  1.0 mm (corresponding to a wavenumber of $k=\frac{2\pi }{\lambda }=6283$). In the absence of surface tension effects, the growth rate of the system due to the Rayleigh‐Taylor instability is given by [48]

(3) $$\sigma =\sqrt{\mathrm{Agk}}\;,$$

where *g*  =  9.81 *m* *s* ^−2^ is the acceleration due to gravity.

### 5.5 Numerical Simulation

Numerical simulations were performed using COMSOL Multiphysics’ laminar flow module. In all laminar flow simulations, fluid properties corresponding to glycerol (μ  =  1.412 Pa s,  ρ  =  1.26 g/cm^3^) were used. For modelling the flow transition in hexagonal and circular cropped array devices, a generic model of each transition region was examined. For the hexagonal array design, the transition region was modelled as a loft with a hexagonal inlet face of major radius 10 mm, a circular outlet face of radius 10 mm, and a length of 100 mm. For the circular‐cropped array design, the transition region was modelled as a cylinder of radius 10 mm and length 100 mm. In both cases, a uniform flow inlet condition with *u*  =  1.00 m/s was applied at the inlet face, and a *P*  =  0 Pa outlet condition was applied at the outlet face. After computation, streamlines were plotted with a uniform density at the inlet face, and the *x* − *y* locations at the inlet and outlet faces were exported. These locations were used to calculate normalised transformation functions for the distortion model as described in Section S4.

For visualization of the 3D pressure fields (Figure 2a–d, Figure S4a–f), the contained fluid volumes for example capillary array designs were exported from Autodesk Fusion 360 in STEP format and imported into COMSOL Multiphysics. Using the laminar flow module, inlet boundary conditions were applied to each inlet proportional to the number of connected capillaries, and a *P*  =  0 Pa outlet condition was applied at the end of the transition region.

A similar approach was used for visualisation of multicompartmental flow in Figure 1c; however, in this case the *laminar flow: transport of chemical species (tcs)* physics module was applied. This approach enabled segmentation of the laminar flow according to fluid inputs, without requiring complex computation of two‐phase fluid interfaces over the rapidly changing length scales in the transition region. To ensure resolution of fine flow features, a diffusion coefficient of 10^−6^ m^2^/s was applied to the physics model, and the mesh was manually refined such that a minimum of 2000 elements were maintained in each of the cross‐sectional slices.

### 5.6 Statistical Analysis

The following analysis applies to measurements of sample viscosities and densities reported in Section S7 and S7.1, respectively.

For the aqueous PVA solutions used in our experiments, measurements of shear stress (*Pa*) were recorded using Anton Paar RheoCompass software, and standard Newtonian viscosity was fitted over a shear rate range of 0 − 500 s^−1^. *n*  =  1 experiments were performed for each of the seven solution concentrations, with standard error (SE) for individual fits ranging in the interval (0.05,  0.34) mPa s (vertical error bars in Figure S7 are too small to discern). Uncertainty in the w/w concentration of aqueous PVA solutions is calculated according to standard uncertainty propagation, taking the baseline precision of  ± 0.0001 g for the digital scale and assuming a maximum uncertainty of ± 0.01 g due to evaporation over the duration of the measurements.

Similarly, we assume that the uncertainty in density measurement of aqueous 5% Alginate and 14.7% PVA samples reported in Section S7.1 is dominated by visual measurement of sample volume in the 15 mL falcon tubes used in our experiments (352096, Corning). In this case, density measurements for both solutions were repeated for *n*  =  3 samples and standard error was calculated according to $SE=\sigma /\sqrt{n}$ for σ the standard deviation of each *n*  =  3 population.

## Conflicts of Interest

The authors declare no conflict of interest.

## Supporting information




**Supporting File 1**: smll74990‐sup‐0001‐SuppMat.docx.

**Supporting File 2**: smll74990‐sup‐0002‐DataFile.zip

**Supporting File 3**: smll74990‐sup‐0003‐VideoS1.mp4.

**Supporting File 4**: smll74990‐sup‐0004‐VideoS2.mp4.

**Supporting File 5**: smll74990‐sup‐0005‐VideoS3.mp4.

**Supporting File 6**: smll74990‐sup‐0006‐VideoS4.mp4

## Data Availability

The data that support the findings of this study are available in the supplementary material of this article.
