# Supplementary material for: Microfluidic Capillary Arrays for Pixelated Control of Multimaterial Flows
Source: Small. 2026 Aug 11;22(56):e08537. doi: 10.1002/smll.202508537 (PMC13653350; doi:10.1002/smll.202508537)
Supplement: Supplementary file 1 [file SMLL-22-e08537-s003.docx]

# **Supporting Information: Microfluidic Capillary Arrays for Pixelated Control of Multimaterial Flows**

Max Chapman^1^, Muhammad Utomo^1^, David J. Collins ^1,2^*

^1^Department of Biomedical Engineering, University of Melbourne, Melbourne, Victoria, Australia

^2^Grame Clarke Institute, University of Melbourne, Parkville, Victoria, Australia

***** Corresponding author

E-mail: [david.collins@unimelb.edu.au](mailto:david.collins@unimelb.edu.au)

Section 1: Pressure field modelling

SI Section 1 describes the mathematical model used to generate the curves in Fig. 2e-f and provides a more general treatment including arbitrary values of the manifold height, $h$.

As reported by Tamayol and Bahrami,^[38]^ the Brinkman equation (Eq. S1) can be applied to estimate the fluid resistance of a confined field of micropillars with permeability $K$ (Eq. S2).

$\frac{\Delta P}{L}=\frac{-\mu Qsinh\left( \frac{h}{\sqrt{\mu^{'}K}} \right)}{Kh\left[ \frac{2\sqrt{\mu^{'}K}}{h}\left[ -1+\cosh\left( \frac{h}{\sqrt{\mu'K}} \right) \right]-\sinh\left( \frac{h}{\sqrt{\mu'K}} \right) \right]}$	(S1)

**Equation S1.** Brinkman equation estimating the pressure drop due to flow across a confined field of pillars, where $Q$ is the volumetric flow rate, $\mu$ the dynamic viscosity of the fluid, $\mu^{'}\approx1/\varepsilon$ the viscosity ratio in the porous region, $K$ the permeability of the porous region,^[38]^ $L$ the length in the direction of flow and $h$ the height of the channel.

$\frac{K}{D^{2}}=\frac{0.16\left[ \frac{\pi}{2\sqrt{3}\varphi}-3\sqrt{\frac{\pi}{2\sqrt{3}\varphi}}+3-\sqrt{\frac{2\sqrt{3}\varphi}{\pi}} \right]}{\sqrt{1-\varphi}}$ ,	(S2)

**Equation S2.** Permeability of a hexagonally-staggered array of pillars, as derived by Tamayol and Bahrami via dimensional analysis.^[38]^ The porosity of the confined region is given by $\varepsilon=1-\varphi$ where the solid volume fraction $\varphi=\frac{\pi D^{2}}{2\sqrt{3}s^{2}}$ for pillar spacing $s$ and pillar diameter $D$, where $D=d+2t$ corresponds to the outer diameter of the capillary structures.

Here, we introduce the relative pillar diameter $\rho=D/s,$ set $L=s$ and substitute Eq. S2 for $K$ in Eq. S1, giving an expression for the pressure drop along the length of a single unit cell of staggered pillars. Setting $h=5 mm$ (typical in our experiments) in Eq. S3 produces the curves given in Fig. 2f.

$\frac{{\Delta P}_{UC}}{\mu Q}=-\frac{6.25\sqrt{1-\frac{\pi\rho^{2}}{2\sqrt{3}}}Sinh\left[ \frac{2.5h}{\sqrt{\frac{s^{2}\rho^{2}\left( 3-3\sqrt{\frac{1}{\rho^{2}}}+\frac{1}{\rho^{2}}-\sqrt{\rho^{2}} \right)}{\left( 1-\frac{\pi\rho^{2}}{2\sqrt{3}} \right)^{3/2}}}} \right]}{hs^{2}\rho^{2}\left( 3-3\sqrt{\frac{1}{\rho^{2}}}+\frac{1}{\rho^{2}}-\sqrt{\rho^{2}} \right)\left( \frac{0.8\sqrt{\frac{s^{2}\rho^{2}\left( 3-3\sqrt{\frac{1}{\rho^{2}}}+\frac{1}{\rho^{2}}-\sqrt{\rho^{2}} \right)}{\left( 1-\frac{\pi\rho^{2}}{2\sqrt{3}} \right)^{3/2}}}\left( -1+Cosh\left[ \frac{2.5h}{\sqrt{\frac{s^{2}\rho^{2}\left( 3-3\sqrt{\frac{1}{\rho^{2}}}+\frac{1}{\rho^{2}}-\sqrt{\rho^{2}} \right)}{\left( 1-\frac{\pi\rho^{2}}{2\sqrt{3}} \right)^{3/2}}}} \right] \right)}{h}-Sinh\left[ \frac{2.5h}{\sqrt{\frac{s^{2}\rho^{2}\left( 3-3\sqrt{\frac{1}{\rho^{2}}}+\frac{1}{\rho^{2}}-\sqrt{\rho^{2}} \right)}{\left( 1-\frac{\pi\rho^{2}}{2\sqrt{3}} \right)^{3/2}}}} \right] \right)}$	(S3)

**Equation S3.** Normalised unit cell pressure $P_{UC}/\mu Q$ as a function relative pillar diameter $\rho,$ pillar spacing $s$ and manifold height, $h$.

We then make the approximation that a ring of unit cells with radially converging flow has equivalent fluid resistance to transverse flow through the same number of unit cells arranged in parallel (Fig S1a). Noting that the hexagonal field of $n$ pillars can be modelled as $m$ concentric rings of unit cells, the pressure drop between the outer edge of the manifold region and the central pixel cluster then is estimated by Equation S4. Fig. 2g gives the estimated maximal pressure drop within the manifold for a given unit cell pressure drop $P_{UC}$ and the total number of array pixels, $n.$

${\Delta P}_{12}=\frac{P_{UC}}{6}\sum_{i=j}^{m} \frac{1}{i} ,$	(S4)

$j=\frac{1}{6}\left( -3+\sqrt{3}\sqrt{-1+4k} \right)$	(S5)

$m=\frac{1}{6}\left( -3+\sqrt{3}\sqrt{-1+4n} \right)$	(S6)

**Equation S4.** Estimated maximum manifold pressure drop $\Delta P_{12}$ for $m$ concentric hexagonal rings of unit cells around a central cluster of $k$ pixels.

Rearranging Eq. S4 gives an expression for the geometric pressure drop scaling factor, $F\left( n,k \right)=\Delta P_{12}/\Delta P_{UC}:$

$F\left( n,k \right)=\frac{1}{6}\sum_{i=j}^{m} \frac{1}{i} ,$	(S7)

$$j=\frac{1}{6}\left( -3+\sqrt{3}\sqrt{-1+4k} \right)$$

$$m=\frac{1}{6}\left( -3+\sqrt{3}\sqrt{-1+4n} \right)$$

**Equation S7.** Pressure drop scaling factor, $F\left( n,k \right)=\Delta P_{12}/\Delta P_{UC}.$

Meanwhile, the minimal capillary pressure drop, $P_{23}$, can be estimated using the Hagen-Poiseuille equation

$\frac{\Delta P_{23}}{\mu Q}=\frac{128l}{\pi kd^{4}}$ ,	(S8)

**Equation S8.** Pressure drop across $k$ capillaries connected to the lowest-level manifold in the array, adapted from the Hagen-Poiseuille equation.

where $l$ is the shortest capillary length, k the number of capillaries enclosed by the field of occluding pillars and $d$ is the internal capillary diameter (Fig. 1a).

When designing a capillary array device, the choice of parameters $d$ and $t$ are governed by practical constraints relating to the minimum feature size of the 3D printer and mechanical robustness of thin printed capillary walls. With these parameters selected and a desired array resolution of $n$ pixels, the pixel spacing, $s$, minimum capillary length, $l$, and manifold height, $h$, should be selected such that Eq. 2 is satisfied.

To illustrate the parameter selection process we highlight the array design shown in Fig. 2a, which has parameters $d=0.50 \mathrm{mm}$, $t=0.25 \mathrm{mm}$ and $s=1.50 \mathrm{mm},$ resulting in a normalised unit cell pressure of $\frac{{\Delta P}_{UC}}{\mu Q}\approx1.25\times{10}^{10}m^{-3}$. Noting that the array is circular-cropped with $n=397$ pixels and a central cluster of $k=19$ pixels, the manifold pressure drop scaling factor given by Fig. 2g is approximately 0.25, such that $\frac{\Delta P_{12}}{\mu Q}\approx3.13\times{10}^{9}m^{-3}$. The chosen value of $l=2.5 \mathrm{mm}$ gives a normalized capillary pressure drop $\frac{\Delta P_{23}}{\mu Q}\approx8.58\times{10}^{10}m^{-3}$, such that the ratio $\frac{\Delta P_{12}}{\Delta P_{23}}\approx0.036.$ This value presents good agreement with the result obtained via numerical simulation (0.038, Fig. 2c), especially noting that the horizontal pressure gradient neglected from the analysis

Further examination was performed to explore the relationship between the array design and the manifold pressure gradients. Fig. S1b shows the influence of varying $k$ on the relative pressure drop ${{\Delta P}_{12}/\Delta P}_{23}$ for the example device shown in Fig. 2a-d, keeping all other design parameters unchanged. Notably, a global maximum exists for $k\approx145$ for the $n=397$ array, suggesting that for a given value of $n$ that a there exists a value $1<k<n$ that results in a maximal relative manifold pressure drop, ${{\Delta P}_{12}/\Delta P}_{23}$. Meanwhile, Fig. S1c shows the difference in manifold pressure drop scaling factor when comparing hexagonal and a circular-cropped arrays for $k=1$, using the amended expression for $m$ given in Eq. S9, below. This difference is small for $n>100$, and consequently was neglected from the primary analysis.

$m=\left\{ \begin{aligned} \frac{1}{6}\left( -3+\sqrt{3}\sqrt{-1+4n} \right), &hexagonal array \\ \sqrt{\frac{\sqrt{3}n}{2\pi}}, &circular-cropped array \end{aligned} \right.$	(S9)

**Figure S1. Additional factors influencing manifold pressure ratio.** (a) Illustration showing similar fluid resistance of 18 micropillar unit cells arranged in a line with transverse flow (left) and in a ring with radial flow (right). Each circle in the diagram represents a unit cell of pillars. (b) Manifold-capillary pressure drop ratio, ${{\Delta P}_{12}/\Delta P}_{23}$, as a function of the central pixel number, $k$, keeping all other design parameters constant (reference design per Fig. 2a-d). (c) Comparison of scaling factor ${{\Delta P}_{12}/\Delta P}_{UC}$ for hexagonal and circular-cropped array configurations with $k=1$, showing negligible difference between both treatments for $n>200$.

Section 2: Intersection over Union Analysis

**Figure S2.** **Intersection over Union (IoU) analysis of experimental flow cross sections.** (a) Experimental images and IoU-overlaid images for Radioactive, Ring, Star and CBML Logo target designs, each with pixel numbers $n=91, 271$ and $469$. The CBML Logo design is reproduced with permission from the Collins BioMicrosystems Laboratory. (b) IoU values as a function of array size, $n$, for all four target geometries.

Experimental images from Figure 3 were compared to their target images using a conventional IoU approach (Equation S10), with IoU value serving as a measure of the accuracy of reproduction of the target geometry.

$IoU= \frac{Target \cap\mathrm{Experiment}}{Target \cup\mathrm{Experiment}}$	(S10)

Images were first segmented using GIMP software via a threshold function, with some manual cleanup required due to image defects or uneven background brightness in the microscope images. To account for misalignment or scaling of the viewing window, each segmented image was iteratively resized and rectilinearly offset until the maximal IoU value was achieved. Maximal IoU values for each design are reported in Fig. S2b.

Section 3: Application & workflow

**Figure S3.** **Automated workflow for generation of extruder geometries.** The Python script “FE Image Plugin.py” takes the input geometry in BMP format (binary or grayscale) and a series of design parameters (pixel number, $n$, number of fluid inputs, $p$, array shape (hexagonal or circular), distortion compensation ON/OFF) and outputs a list of normalised pixel coordinates for each fluid input. With the parametric template model open in Autodesk Fusion 360, running the Python script “FE API Plugin.py” takes the output of the previous script and a series of geometric parameters (capillary diameter, $d$, capillary spacing, $s$, wall thickness, $t$, minimum capillary length, $l$, and manifold height, $h$) and automatically builds the capillary array geometry. The model can then be exported in .STL or .STEP format for 3D printing. The CBML Logo design is reproduced with permission from the Collins BioMicrosystems Laboratory.

Section 4: Experimental setup

**Figure S4**. **Experimental setup for in-flow imaging of dual-fluid flow.** (a) Schematic diagram depicting in-flow imaging setup components. (b) Photograph of example setup used for in-flow imaging experiments, with pure glycerol and 0.1% w/w blue food dye in glycerol as the fluid inputs.

Section 5: Design improvements

**Figure S5. Radial diffuser design validation**. (a-f) Numerical simulations for comparison of pressure fields within lowermost manifold region for a four-fluid extruder device without (a-c) and with (d-f) a radial diffuser feature. The inclusion of a radial diffuser significantly diminishes the horizontal component of the pressure gradient, as evidenced by the 3.80% and 0.42% pressure disparities along the extremes of Cut lines 1 (c) and 2 (f) respectively. (g) Time series demonstrating the perfusion of fluid through the radial diffuser with quasi-radial symmetry. Prior to t=0, the manifold was primed with clear glycerol. At t=0, blue-dyed glycerol was injected into the manifold chamber.

Section 6: Distortion modelling

**Figure S6.** **Distortion model for transition region between undeveloped flow and fully developed Poiseuille flow**. (a) Section view showing the locations for coordinate spaces $(x_{1},y_{1})$ and $(x_{2},y_{2})$. (b-c) Surface plots showing the forward transformation functions $x_{2}(x_{1},y_{1})$ and $y_{2}\left( x_{1},y_{1} \right)$ for a hexagonal capillary array. (d-e) Surface plots showing the forward transformation functions $x_{2}(x_{1},y_{1})$ and $y_{2}\left( x_{1},y_{1} \right)$ for a circular-cropped capillary array. n=12 polynomial functions for (b-e) are given in 4.1.

6.1 Transformation functions

Simulation results for the flow transition region were approximated using 12^th^-order polynomial fits for hexagonal and circular-cropped arrays.

**Hexagonal Array:**

$x_{2,h}\left( x_{1},y_{1} \right)= 0.78954x+0.00032338x^{2}+1.7683x^{3}-0.052746x^{4}-43.444x^{5}+1.5756x^{6}+487.97x^{7}-17.083x^{8}-2323.1x^{9}+78.833x^{10}+4055.3x^{11}-130.14x^{12}-0.00093083y+0.00043635xy+0.053794x^{2}y+0.087482x^{3}y+0.016502x^{4}y-3.5369x^{5}y-17.155x^{6}y+53.732x^{7}y+172.83x^{8}y-347.26x^{9}y-470.83x^{10}y+783.78x^{11}y+0.0040624y^{2}+1.7189xy^{2}+0.2732x^{2}y^{2}-159.59x^{3}y^{2}-11.249x^{4}y^{2}+4983.7x^{5}y^{2}+169.16x^{6}y^{2}-43568.x^{7}y^{2}-1103.7x^{8}y^{2}+122660.x^{9}y^{2}+2533.8x^{10}y^{2}+0.080847y^{3}-0.0995xy^{3}-5.7257x^{2}y^{3}+0.25274x^{3}y^{3}+79.186x^{4}y^{3}+18.704x^{5}y^{3}-380.47x^{6}y^{3}-247.96x^{7}y^{3}+454.47x^{8}y^{3}+1080.9x^{9}y^{3}-0.27755y^{4}-16.725xy^{4}-4.103x^{2}y^{4}+2004.9x^{3}y^{4}+140.37x^{4}y^{4}-47499.x^{5}y^{4}-1334.1x^{6}y^{4}+230070.x^{7}y^{4}+4212.9x^{8}y^{4}-1.8868y^{5}+3.6275xy^{5}+102.36x^{2}y^{5}-27.58x^{3}y^{5}-964.92x^{4}y^{5}+88.941x^{5}y^{5}+2678.9x^{6}y^{5}-615.91x^{7}y^{5}+6.0717y^{6}-126.23xy^{6}+10.668x^{2}y^{6}-4736.6x^{3}y^{6}-609.06x^{4}y^{6}+105490.x^{5}y^{6}+2812.4x^{6}y^{6}+18.561y^{7}-46.729xy^{7}-658.75x^{2}y^{7}+252.78x^{3}y^{7}+2951.x^{4}y^{7}-93.412x^{5}y^{7}-58.039y^{8}+1456.4xy^{8}+92.835x^{2}y^{8}-8560.1x^{3}y^{8}+866.57x^{4}y^{8}-82.296y^{9}+244.62xy^{9}+1420.6x^{2}y^{9}-760.2x^{3}y^{9}+252.28y^{10}-2563.7xy^{10}-388.96x^{2}y^{10}+135.65y^{11}-443.7xy^{11}-408.56y^{12}$

$y_{2,h}\left( x_{1},y_{1} \right)= 0.00048464x-0.00021324x^{2}-0.046054x^{3}+0.0038508x^{4}+1.4233x^{5}+0.17439x^{6}-17.716x^{7}-3.406x^{8}+94.89x^{9}+18.763x^{10}-182.33x^{11}-32.18x^{12}+0.78926y-0.0024138xy+1.7136x^{2}y+0.37086x^{3}y-78.49x^{4}y-13.576x^{5}y+1565.1x^{6}y+189.02x^{7}y-10562.x^{8}y-1112.4x^{9}y+23710.x^{10}y+2327.7x^{11}y-0.0054877y^{2}-0.10207xy^{2}+0.74694x^{2}y^{2}+4.9399x^{3}y^{2}-35.682x^{4}y^{2}-97.076x^{5}y^{2}+591.74x^{6}y^{2}+761.28x^{7}y^{2}-3905.1x^{8}y^{2}-1963.1x^{9}y^{2}+8842.3x^{10}y^{2}+1.8061y^{3}+0.029778xy^{3}-35.463x^{2}y^{3}-4.9186x^{3}y^{3}+2361.x^{4}y^{3}+151.17x^{5}y^{3}-31985.x^{6}y^{3}-1433.5x^{7}y^{3}+115000.x^{8}y^{3}+4248.6x^{9}y^{3}+0.17107y^{4}+3.1408xy^{4}-8.7375x^{2}y^{4}-77.947x^{3}y^{4}+385.59x^{4}y^{4}+824.09x^{5}y^{4}-4752.5x^{6}y^{4}-3168.3x^{7}y^{4}+16678.x^{8}y^{4}-57.243y^{5}-0.88087xy^{5}-539.89x^{2}y^{5}+38.746x^{3}y^{5}-8016.3x^{4}y^{5}-694.1x^{5}y^{5}+108490.x^{6}y^{5}+3018.8x^{7}y^{5}-2.9107y^{6}-39.171xy^{6}+15.072x^{2}y^{6}+500.56x^{3}y^{6}-1045.3x^{4}y^{6}-1861.4x^{5}y^{6}+8295.x^{6}y^{6}+1022.4y^{7}+13.333xy^{7}+6029.1x^{2}y^{7}-228.95x^{3}y^{7}-14859.x^{4}y^{7}+1566.1x^{5}y^{7}+28.418y^{8}+213.86xy^{8}+104.2x^{2}y^{8}-1257.1x^{3}y^{8}+190.55x^{4}y^{8}-7173.7y^{9}-67.103xy^{9}-13818.x^{2}y^{9}+463.83x^{3}y^{9}-140.87y^{10}-411.27xy^{10}-254.07x^{2}y^{10}+17854.y^{11}+109.98xy^{11}+269.79y^{12}$

**Circular-cropped Array:**

$x_{2,c}\left( x_{1},y_{1} \right)= 0.72264x_{1}-0.0046686x_{1}^{2}+1.7558x_{1}^{3}+0.0045141x_{1}^{4}-38.086x_{1}^{5}+1.1387x_{1}^{6}+410.43x_{1}^{7}-12.243x_{1}^{8}-1904.x_{1}^{9}+48.892x_{1}^{10}+3288.7x_{1}^{11}-68.659x_{1}^{12}-0.00013433y_{1}+0.00912x_{1}y_{1}-0.064194x_{1}^{2}y_{1}-0.14427x_{1}^{3}y_{1}+1.3144x_{1}^{4}y_{1}+0.30893x_{1}^{5}y_{1}-9.3628x_{1}^{6}y_{1}+3.5893x_{1}^{7}y_{1}+28.137x_{1}^{8}y_{1}-15.841x_{1}^{9}y_{1}-29.767x_{1}^{10}y_{1}+12.55x_{1}^{11}y_{1}-0.0032495y_{1}^{2}+1.7959x_{1}y_{1}^{2}+0.9508x_{1}^{2}y_{1}^{2}-73.521x_{1}^{3}y_{1}^{2}-13.262x_{1}^{4}y_{1}^{2}+1193.3x_{1}^{5}y_{1}^{2}+59.68x_{1}^{6}y_{1}^{2}-7469.x_{1}^{7}y_{1}^{2}-74.299x_{1}^{8}y_{1}^{2}+16287.x_{1}^{9}y_{1}^{2}-65.809x_{1}^{10}y_{1}^{2}+0.013122y_{1}^{3}-0.66293x_{1}y_{1}^{3}+2.5979x_{1}^{2}y_{1}^{3}+13.909x_{1}^{3}y_{1}^{3}-40.543x_{1}^{4}y_{1}^{3}-81.626x_{1}^{5}y_{1}^{3}+196.93x_{1}^{6}y_{1}^{3}+121.47x_{1}^{7}y_{1}^{3}-311.48x_{1}^{8}y_{1}^{3}+93.074x_{1}^{9}y_{1}^{3}+0.0016306y_{1}^{4}-39.243x_{1}y_{1}^{4}-24.109x_{1}^{2}y_{1}^{4}+1189.x_{1}^{3}y_{1}^{4}+288.17x_{1}^{4}y_{1}^{4}-10909.x_{1}^{5}y_{1}^{4}-1073.5x_{1}^{6}y_{1}^{4}+31701.x_{1}^{7}y_{1}^{4}+1252.3x_{1}^{8}y_{1}^{4}-0.45154y_{1}^{5}+12.036x_{1}y_{1}^{5}-30.531x_{1}^{2}y_{1}^{5}-229.16x_{1}^{3}y_{1}^{5}+322.92x_{1}^{4}y_{1}^{5}+1174.8x_{1}^{5}y_{1}^{5}-782.21x_{1}^{6}y_{1}^{5}-1597.4x_{1}^{7}y_{1}^{5}+1.1266y_{1}^{6}+424.96x_{1}y_{1}^{6}+226.9x_{1}^{2}y_{1}^{6}-7449.2x_{1}^{3}y_{1}^{6}-1946.5x_{1}^{4}y_{1}^{6}+31357.x_{1}^{5}y_{1}^{6}+3918.4x_{1}^{6}y_{1}^{6}+5.3252y_{1}^{7}-88.678x_{1}y_{1}^{7}+137.47x_{1}^{2}y_{1}^{7}+1219.9x_{1}^{3}y_{1}^{7}-746.42x_{1}^{4}y_{1}^{7}-3567.5x_{1}^{5}y_{1}^{7}-13.681y_{1}^{8}-1980.8x_{1}y_{1}^{8}-901.4x_{1}^{2}y_{1}^{8}+16373.x_{1}^{3}y_{1}^{8}+4081.1x_{1}^{4}y_{1}^{8}-25.045y_{1}^{9}+292.36x_{1}y_{1}^{9}-209.37x_{1}^{2}y_{1}^{9}-2041.8x_{1}^{3}y_{1}^{9}+60.458y_{1}^{10}+3423.2x_{1}y_{1}^{10}+1272.6x_{1}^{2}y_{1}^{10}+40.943y_{1}^{11}-361.09x_{1}y_{1}^{11}-92.404y_{1}^{12}$

$y_{2,c}\left( x_{1},y_{1} \right)=0.00051281x_{1}+0.0026108x_{1}^{2}+0.017849x_{1}^{3}-0.08051x_{1}^{4}-0.30121x_{1}^{5}+0.60886x_{1}^{6}+2.6924x_{1}^{7}-0.28501x_{1}^{8}-11.473x_{1}^{9}-10.653x_{1}^{10}+18.085x_{1}^{11}+26.1x_{1}^{12}+0.7222y_{1}-0.0068652x_{1}y_{1}+1.8241x_{1}^{2}y_{1}+0.67986x_{1}^{3}y_{1}-39.549x_{1}^{4}y_{1}-16.15x_{1}^{5}y_{1}+425.58x_{1}^{6}y_{1}+151.09x_{1}^{7}y_{1}-1978.1x_{1}^{8}y_{1}-616.09x_{1}^{9}y_{1}+3416.7x_{1}^{10}y_{1}+918.8x_{1}^{11}y_{1}-0.0022996y_{1}^{2}+0.027026x_{1}y_{1}^{2}-0.28991x_{1}^{2}y_{1}^{2}-0.21054x_{1}^{3}y_{1}^{2}+13.517x_{1}^{4}y_{1}^{2}-4.0174x_{1}^{5}y_{1}^{2}-163.46x_{1}^{6}y_{1}^{2}+38.071x_{1}^{7}y_{1}^{2}+745.69x_{1}^{8}y_{1}^{2}-80.443x_{1}^{9}y_{1}^{2}-1148.9x_{1}^{10}y_{1}^{2}+1.7561y_{1}^{3}-0.11024x_{1}y_{1}^{3}-73.825x_{1}^{2}y_{1}^{3}-0.46178x_{1}^{3}y_{1}^{3}+1188.5x_{1}^{4}y_{1}^{3}+71.461x_{1}^{5}y_{1}^{3}-7407.x_{1}^{6}y_{1}^{3}-610.61x_{1}^{7}y_{1}^{3}+16212.x_{1}^{8}y_{1}^{3}+1364.3x_{1}^{9}y_{1}^{3}+0.072891y_{1}^{4}-1.1155x_{1}y_{1}^{4}-0.055798x_{1}^{2}y_{1}^{4}+16.6x_{1}^{3}y_{1}^{4}-84.595x_{1}^{4}y_{1}^{4}-45.946x_{1}^{5}y_{1}^{4}+965.43x_{1}^{6}y_{1}^{4}-29.554x_{1}^{7}y_{1}^{4}-2630.4x_{1}^{8}y_{1}^{4}-38.083y_{1}^{5}+3.178x_{1}y_{1}^{5}+1198.2x_{1}^{2}y_{1}^{5}-67.709x_{1}^{3}y_{1}^{5}-10934.x_{1}^{4}y_{1}^{5}+184.26x_{1}^{5}y_{1}^{5}+31335.x_{1}^{6}y_{1}^{5}+394.77x_{1}^{7}y_{1}^{5}-0.70981y_{1}^{6}+11.889x_{1}y_{1}^{6}+28.18x_{1}^{2}y_{1}^{6}-148.59x_{1}^{3}y_{1}^{6}-62.15x_{1}^{4}y_{1}^{6}+341.08x_{1}^{5}y_{1}^{6}-743.63x_{1}^{6}y_{1}^{6}+410.74y_{1}^{7}-25.824x_{1}y_{1}^{7}-7505.9x_{1}^{2}y_{1}^{7}+449.1x_{1}^{3}y_{1}^{7}+31835.x_{1}^{4}y_{1}^{7}-1185.6x_{1}^{5}y_{1}^{7}+3.2449y_{1}^{8}-46.139x_{1}y_{1}^{8}-133.35x_{1}^{2}y_{1}^{8}+335.06x_{1}^{3}y_{1}^{8}+582.8x_{1}^{4}y_{1}^{8}-1906.y_{1}^{9}+88.415x_{1}y_{1}^{9}+16374.x_{1}^{2}y_{1}^{9}-805.16x_{1}^{3}y_{1}^{9}-8.7225y_{1}^{10}+57.542x_{1}y_{1}^{10}+180.43x_{1}^{2}y_{1}^{10}+3291.4y_{1}^{11}-112.07x_{1}y_{1}^{11}+12.09y_{1}^{12}$

Section 7: Additional Fluid Flow Properties

7.1 PVA/Alginate viscosity matching

**Figure S7. Viscosity matching of PVA and sodium alginate solutions.** (a) Viscosity curve of sodium alginate parent solution used for all crosslinked extrusions. The 100-500$s^{-1}$ region of interest (ROI) corresponding to the flow transition region of a typical device is marked in grey, and the average viscosity of the solution in this region is 194.4 mPa s. (b) Viscosity of aqueous PVA solutions with increasing concentration from 13-19% w/w. The ROI-averaged alginate viscosity corresponds to a PVA concentration of approximately 14.7%. Each point in (b) represents an n=1 rheology experiment with standard error in the range $(0.05, 0.34) mPa s$ (not shown) and horizontal error bars representing standard uncertainty in w/w density measurements.

7.2 Brief investigation of the Rayleigh-Taylor Instability (RTI)

Where the matching of fluid viscosities is a critical feature of our technique, specific efforts to match the of co-flowing fluids were omitted from our experimental design. This choice is justified by several assumptions regarding to our setup, namely:

(1) Density differences between co-flowing fluids are small (or in the case of glycerol-glycerol + food dye fluid combinations, negligible);
(2) Accelerations applied to the fluid in the microfluidic system are small;
(3) The transit time of the fluid through the flow transition region is small when compared with the RTI growth rate, $\gamma$.

Below, we validate each of these assumptions in relation to the experimental parameters reported in our manuscript. It should be noted that, if our technique were to be applied with significantly different fluid properties or length scales, these assumptions would need to be reassessed.

7.2.1 Density disparity of experimental fluids

Due to the low concentration of food dye (0.1% w/w), there was no measurable difference in the density of our experimental glycerol and glycerol + food dye solutions. As such, the following RTI analysis applies for the coflowing aqueous alginate + PVA solutions only. The aqueous 5% Sodium Alginate and 14.7% PVA solutions, meanwhile, exhibit a small but measurable density contrast. Density measurements were obtained by pipetting $10 mL$ aliquots of each solution into test tubes on a Kern Analytical Balance ABS 320-4N precision scale ($d=0.1 mg$). The weight of each 10mL sample was averaged over $n=3$ repeats, resulting in measured densities of $\rho_{1}=1.035\pm0.008 g/cm^{3}$ for the 5% aqueous sodium alginate and $\rho_{2}=1.045\pm0.015 g/cm^{3}$ for the 14.7% aqueous PVA solution (mean $\pm1.96\times SE$, 95% confidence interval).

In conventional analyses of the RTI, the density contrast of a fluid interface is characterised by the Atwood number, $A$, given by Eq. S11:

$A=\frac{\rho_{2}-\rho_{1}}{\rho_{2}+\rho_{1}}$,	(S11)

Where $\rho_{2}$ and $\rho_{1}$ are the densities of the fluids at either side of the interface, with $\rho_{2}>\rho_{`1}$. Taking the extremes of each 95% confidence interval in our measured densities ($\rho_{1}=1.027, \rho_{2}=1.060$), the maximum Atwood number evaluates to approximately $A\approx0.016$.

7.2.2 Fluid acceleration in the flow transition region

Here we calculate the average acceleration applied to a infinitesimal volume of fluid transiting the flow transition region, with device parameters $d_{1}=50.0 mm$ and $d_{2}=1.5 mm$ describing the diameters of the start and end of the transition region, respectively, and $L=25.0 mm$ the length of the transition region parallel to the direction of flow. These values represent the most heavily-converging flow investigated in our experiments. Taking a total combined flow rate of $Q=10 mL/min$ (typical for a device of this size), the average velocities at the start ($u$) and end ($v$) of the transition region are given below:

$$\bar{u}=\frac{4Q}{\pi d_{1}^{2}} \approx8.5\times{10}^{-5} m/s$$

$$\bar{v}=\frac{4Q}{\pi d_{2}^{2}} \approx2.1\times{10}^{-1} m/s$$

Noting that the flow profile at the beginning of the transition is approximately uniform, $u_{min}=u_{max}=\bar{u}$, while for the fully-developed Poiseuille flow at the end of the transition region, $v_{max}=2\times\bar{v}=4.2\times{10}^{-1} m/s$. Approximating the acceleration between the beginning and end of the transition regions as constant, we can estimate the average and maximum acceleration experienced by an infinitesimal volume of fluid, with the peak acceleration occurring along the centreline of the flow:

$$\bar{a}=\left( \bar{v}^{2}-\bar{u}^{2} \right)/(2L)\approx0.90 m/s^{2}$$

$$a_{max}=\left( v_{max}^{2}-u_{min}^{2} \right)/(2L)\approx3.60 m/s^{2}$$

Substituting these values into the first equation of motion allows estimation of the average and minimum transit times for fluid in the flow transition region:

$$\bar{t}=\frac{\left( \bar{v}-\bar{u} \right)}{\bar{a}}\approx0.24 s$$

$$t_{min}=\frac{\left( v_{max}-u_{min} \right)}{a_{max}}\approx0.12 s$$

Noting that the maximum acceleration in our system is considerably smaller than the acceleration due to gravity, the ensuing RTI analysis will take the value $g=9.81 m/s^{2}$ as the critical acceleration term in the stability of perturbations at the fluidic interface.

7.2.3 Approximate evaluation of the peak RTI growth rate, $\gamma_{peak}$.

While the classical inviscid model of the RTI describes the growth rate, $\gamma$, as increasing infinitely with the wavenumber, $k$, of perturbations, more recent analysis has shown that the consideration of surface tension and/or viscosity introduces a peak growth rate, $\gamma_{peak},$for finite $k_{peak},$ above which the growth rate tends to zero as $k\to\infty$. As outlined by Mikaelian,^[48]^ for systems such as ours that involve significant viscosity and negligible surface tension at the fluidic interface, the peak RTI growth rate occurs at the below values of $k_{peak}, \gamma_{peak}$:

$k_{peak}=\frac{1}{2}\left( \frac{gA}{\nu^{2}} \right)^{\frac{1}{3}}$	(S12)

$\gamma_{peak}=\frac{1}{2}\left( \frac{g^{2}A^{2}}{\nu} \right)^{\frac{1}{3}}$	(S13)

Where $\nu=(\mu_{2}+\mu_{1})/(\rho_{2}+\rho_{1})$describes the relative viscosity of the fluid mixture for $\mu_{1}$ and $\mu_{2}$ the dynamic viscosities of the lighter and heavier fluids respectively (in our system, $\mu_{1}=\mu_{2}=194.4 mPa s$). Substituting values for $g,A, \mu_{1,2}$ and the extreme cases for $\rho_{1,2}$ into Eq. (12) and (13) gives peak values $k_{peak}\approx0.82 m^{-1}$ and $\gamma_{peak}\approx0.25 s^{-1}.$ Here, we highlight that the average transit time of fluid in the transition of approximately $\bar{t}=0.24 s$, is insufficient for the RTI to significantly impact our system ($\gamma_{peak}\times\bar{t}\approx0.06$).

7.3 Peclet number analysis

The following analysis provides an approximate quantification of the Peclet number for co-flowing streams in a typical flow transition region, with reference to Figure 2e.

The diffusion coefficients of aqueous food dye solutions were not directly measured in our experiments, however literature reports that such dyes likely exhibit diffusion coefficients of less than ${10}^{-10} m^{2}/s$ in pure glycerol.^[36]^ For a typical in-flow imaging experiment using the device shown in Figure 2e, with parameters $n\approx300, s=1 mm, Q=1 mL/min,$ array diameter $d_{3}=18.0 mm$ and outlet diameter $d_{4}=1.0 mm$, we examine two extreme cases characterizing flow at the beginning (3) and end (4) of the flow transition region. In both cases we take the characteristic length as the size of one fluidic pixel.

(3) Immediately downstream of the capillary array:

$$L_{3}=s=1\times{10}^{-3} m$$

$$u_{3}=4Q/(\pi d_{3}^{2})=7\times{10}^{-5}m/s$$

$$Pe_{L,3}=\frac{L_{1}u_{1}}{D}\approx{7\times10}^{2}$$

(4) At the device outlet (noting that pixel size and flow velocity are spatially variable, average both values over the outlet cross section):

$$\bar{L_{4}}=L_{1}\times\frac{d_{4}}{d_{3}}=6\times{10}^{-5} m$$

$$\bar{u_{4}}=4Q/(\pi d_{2}^{2})=2\times{10}^{-2} m$$

$${Pe}_{L,4}=\frac{L_{4}u_{4}}{D}\approx{1\times10}^{4}$$

Noting that ${Pe}_{L}\gg1$ in both cases, this analysis supports our observation of negligible diffusion during in-flow imaging experiments when applying our approach with glycerol and food dye mixtures.

Section 8: CAD models and Python Code

**FE Template Model_Hex.f3d.** Parametric CAD model containing capillary array template, for use with hexagonally-cropped array geometries. With the model open Autodesk Fusion 360, adjusting parameters in the parameter table automatically updates the model geometry. Running FE Plugin.py from the script utility automatically updates the model parameters to the values selected in the script.

**FE Template Model_Round.f3d.** Parametric CAD model containing capillary array template, for use with circular-cropped array geometries. With the model open Autodesk Fusion 360, adjusting parameters in the parameter table automatically updates the model geometry. Running FE Plugin.py from the script utility automatically updates the model parameters to the values selected in the script.

**FE Image Sampler.py.** Python script for postprocessing of .PNG images. This script takes an input .PNG file and samples the image according to the user’s desired parameters (number of pixels, number of fluid inputs, array shape and distortion compensation ON/OFF). The script outputs a list containing sampling parameter information and the normalised coordinates of capillaries for each fluid mapping in the array.

**FE Plugin.zip.** Zip file containing Python script for use with Autodesk Fusion 360’s API. This script accepts a list input as output from FE Image Sampler.py and a set of geometric parameters describing the capillary and manifold dimensions. With the template model open in Autodesk Fusion 360, running FE Plugin.py automatically builds the model according to the stored parameters.

**Radioactive.bmp.** Example bmp image used to generate array coordinates for “Radioactive” geometry in Figure 3a.

**Ring.bmp.** Example bmp image used to generate array coordinates for “Ring” geometry in Figure 3a.

**Star.bmp.** Example bmp image used to generate array coordinates for “Star” geometry in Figure 3a.

**CBML.bmp.** Example bmp image used to generate array coordinates for “CBML Logo” geometry in Figure 3a. Reproduced with permission from the Collins BioMicrosystems Laboratory.

**Microfibre.bmp**. Example bmp image used to generate array coordinates for “Microfibre” geometry in Figure 4c.

Section 9: Supplementary Videos

**Supplementary Video 1.** **Perfusion of crosslinked dual-lumen soft microfibre structure.** The alginate microfibre was primed with dry artists’ pigment (Langridge Colours, Australia) mixed in DI water to display fluid motion. Flow was generated by hydrostatic elevation of one end of the soft microfibre.

**Supplementary Video 2.** **Real-time flow evolution of 91-pixel “Radioactive” design.** The flow cross-section was recorded using the in-flow imaging approach described in *Methods*. Intermittent air bubbles can be seen to temporarily disrupt the flow; however, these are eventually cleared from the system after which the steady-state cross-sectional geometry remains fixed.

**Supplementary Video 3.** **Microfluidic wet spinning of alginate microfibres.** The capillary array device outlet is connected to a microfluidic co-flow extrusion chip, in which the pixellated alginate flow cross section is encapsulated by a sheath flow of 10% w/v aqueous calcium chloride (CaCl_2_) solution. The outlet of the microfluidic co-flow extrusion chip is submerged in a bath of 10% w/v CaCl_2_.

**Supplementary Video 4.** **Automated design of example capillary array device.** This screen recording shows the parametrically assembled of an example device’s geometry using the template model *(FE Template Model.f3d)* and Fusion 360 API plugin *(FE Plugin.py).*
